# Editorial: Tooth Enamel: Frontiers in Mineral Chemistry and Biochemistry, Integrative Cell Biology and Genetics

**DOI:** 10.3389/fphys.2018.01153

**Published:** 2018-08-28

**Authors:** Steven J. Brookes, Ariane Berdal, Alexandre R. Vieira, Sylvie Babajko, Jennifer Kirkham

**Affiliations:** ^1^Oral Biology, School of Dentistry, Faculty of Medicine and Health, University of Leeds, Leeds, United Kingdom; ^2^INSERM U1138 Centre de Recherche des Cordeliers, Paris, France; ^3^School of Dental Medicine, University of Pittsburgh, Pittsburgh, PA, United States

**Keywords:** amelogenesis, enamel (mature and developing), enamel pathology, enamel 9 symposium, ameloblast

This Frontiers research topic, Tooth Enamel: Frontiers in Mineral Chemistry and Biochemistry, Integrative Cell Biology and Genetics, was hosted under the Craniofacial Biology and Dental Research speciality. The speciality covers cellular and molecular aspects of the development, pathology, and repair or regeneration of craniofacial tissues and organs; each and all of these aspects are covered in this topic as they relate to dental enamel. The topic was initially conceived as the vehicle by which manuscripts arising from the Enamel 9 International Symposium (held in November 2016 at Rudding Park, Harrogate, UK) would be disseminated but the topic was also open to all enamel researchers to contribute their findings irrespective of their attendance at the Symposium.

The topic presents 34 manuscripts from 15 different countries that together convey the breadth and scope of enamel research across the globe. The research themes of the Enamel 9 Symposium provided the framework on which the topic was based. The themes comprise: (i) Enamel Formation: Stem Cells and Differentiation, (ii) Cell Biology of Amelogenesis, (iii) Enamel Matrix Proteins, (v) Enamel Biomineralization & Biomimetics, (vi) Enamel Evolution and Development, (vii) Enamel Pathology, (viii) Amelogenesis Imperfecta (AI), (ix) Caries, and (x) Animal Models in Enamel Research. The articles are mostly in the form of original research papers but accepted papers also included reviews, perspectives, opinion pieces, and methodological papers.

It is impossible, in this short editorial, to highlight each of the papers submitted to this topic, which span the full breadth of enamel research. We therefore have chosen to take an objective approach and highlight here the top three *original research papers* with the most viewings at the time of writing, along with the most viewed *review, perspective, opinion piece*, and *methodological paper*. Stakkestad et al. report how different ameloblastin processing products, and splice variants thereof, might influence gene expression and proliferation in human mesenchymal stem cells in an autocrine fashion, highlighting the increasing interest in tissue regeneration and stem cell biology amongst enamel researchers. Focusing on amelogenesis imperfecta (AI), Lignon et al. report on the detailed characterization of enamel in enamel renal syndrome caused by a *FAM20A* mutation and conclude that while initial enamel formation is unaffected by the mutation, subsequently secreted enamel is abnormal. The authors discuss the pathological etiology involved by correlating the observed phenotype to loss of FAM20A function. The paper exemplifies the need to move from the phenomenological to the mechanistic if we are to further our knowledge base of the etiology of inherited conditions. The role of amelogenin phosphorylation in enamel biomineralization has long been debated. Yamazaki et al., investigating the role of amelogenin phosphorylation in enamel matrix function, report data indicating that amelogenin (LRAP) phosphorylation on serine 16 influences protein secondary structure and enhances the ability of amelogenin to stabilize mineral precursor phases. It is clear that future functionality work using recombinant amelogenin, for example, must take this post-translational modification in to account. The global research effort to further our understanding of amelogeneisis imperfecta has contributed towards our knowledge of the fundamental biology of enamel biomineralization and will increasingly inform clinical treatment of those affected. Smith et al. review the genes and mutations underlying non-syndromic AI and present a Leiden Open Source Variation Database (LOVD) used to identify trends in genes and mutations causing AI in some 270 families, together with a discussion of how translation of AI genetics can benefit patient care. Molar hypomineralization (MH) is a “silent public health problem” on a global scale, affecting the dental health of up to 1 in 6 children. Hubbard et al. provide a state-of-the-art perspective on MH and argue the need for awareness building and a greater collaborative research effort to tackle this emerging issue while Babajko et al. provide an opinion piece on the potential role of environmental endocrine disrupting chemicals in MH etiology through disruption of endocrine signaling via ameloblast steroid receptors. Finally, the technical aspects of amelogenin research are still of high interest to our research community, as evidenced by those accessing the report by Gabe et al. on the use of a preparative polyacrylamide electrophoretic methodology for amelogenin protein purification.

Space precludes a wider description of the articles published but readers are urged to peruse the on line contents list to view and quickly access the full range of topic articles published. We are confident that the contents will be of interest to all enamel and biomineralization researchers. Clinicians will find up-to-date thinking and opinion on the etiology of enamel pathologies and their potential future treatment via novel strategies for preventing, repairing and even regenerating enamel. Industry-based colleagues will have access the latest advances in translational enamel research.

## Looking to the future

Proceedings arising from the Enamel Symposia reach back over 50 years of enamel research and provide a rich archive offering access to state of the art understanding of all things enamel at that point in time. We are confident that the manuscripts within this research topic continue that tradition, illustrating our current understanding of enamel development, structure and pathology and signposting the future direction of enamel research. The final session of the Enamel 9 Symposium comprised an open forum where enamel researchers from across the world came together to discuss the future priorities and directions for enamel research, identifying the key questions remaining to be answered and highlighting the methodological, technical and clinical challenges that need to be addressed. These views were captured and are presented in an opinion article (Kirkham et al.) published alongside the other articles comprising the topic. Key points raised included: a call for stronger outcomes through interdisciplinary, integrative and translational approaches, a call for a better standardization of experimental variables, better collaboration for a stronger collective voice and outputs and increased and more effective communication. This was a very positive message from the enamel research community and indicates a genuine desire to increase collaboration for the benefit of advancing the field. Increased cross-collaboration between research groups can be challenging given the competition for increasingly scarce funding but advancement of our science and the solving of complex problems demands that we cross disciplinary and geographical boundaries to work together. We look forward to the Enamel 10 Symposium to evidence how enhanced collaboration and interdisciplinarity has shaped enamel research in the intervening period since Enamel 9.

This is the first time that an Enamel Symposium has been associated with Frontiers Media SA and we thank the team at Frontiers for their help with the process, bringing with it the benefits of full open access to the papers as soon as they are accepted for publication following full peer review. We are especially grateful to Thimios Mitsiadis who, as Specialty Chief Editor for Craniofacial Biology and Dental Research, provided invaluable support and advice throughout.

Finally, we owe a huge thank you to all the contributors whose hard work and dedication to their science brought this research topic to fruition. We are all waiting eagerly to see how the research presented here continues to develop and advances our understanding of this amazing tissue: dental enamel.

## Author contributions

All authors listed have made a substantial, direct and intellectual contribution to the work, and approved it for publication.

### Conflict of interest statement

The authors declare that the research was conducted in the absence of any commercial or financial relationships that could be construed as a potential conflict of interest.

